# The cost of treating selected psychiatric conditions from the provider’s perspective in a public tertiary academic hospital in South Africa

**DOI:** 10.3389/fpubh.2026.1954891

**Published:** 2026-10-30

**Authors:** B. Nechombo, M. Sibanda, L. Makhele, M. Matlala

**Affiliations:** 1 Department of Public Health Pharmacy and Management, School of Pharmacy, Sefako Makgatho Health Sciences University, Pretoria, South Africa; 2 Government Employees Medical Scheme, Pretoria, South Africa

**Keywords:** access to medicines, cost of illness, health economics, mental health, non-communicable diseases, psychiatric disorders, South Africa, Sub-Saharan Africa

## Abstract

**Background and objectives:**

Psychiatric disorders share the chronicity and lifelong treatment burden that make the major non-communicable diseases (NCDs) costly to health systems, yet remain comparatively under-costed within the Sub-Saharan African literature on chronic disease burden. Determining the direct costs of treating psychiatric disorders in resource limited settings is important for budget allocation, particularly as South Africa moves towards the National Health Insurance (NHI) fund system. The study aimed to determine the costs of psychiatric outpatient care at a public tertiary hospital in South Africa.

**Methods:**

The study employed a retrospective quantitative approach, using data from patient files at a public tertiary hospital in Gauteng. Data were captured in Microsoft Excel^TM^ and analysed in SPSS®. Direct medical costs were assessed using a micro-costing method to determine the yearly, average total cost of care per psychiatric patient. Ethics approval (SMUREC/P/411/2023: PG) and permission to conduct the study were obtained.

**Results:**

The study reviewed 278 patient files and found a significant relationship (*p* < 0.001) between psychiatric diagnoses and treatment costs. Schizophrenia had the highest average annual cost at R7,248.25 per patient, followed by bipolar disorder at R5,762.10, anxiety disorders at R4,853.14, and depression at R4,792.79. Treatment costs for adverse effects were generally low, except for schizophrenia. Supportive care therapies, such as cognitive behavioural therapy, added moderate expenses, while psychiatric medications were the main cost drivers.

**Conclusion:**

The results highlight the necessity for South African public healthcare systems to allocate resources specifically for the treatment of psychiatric conditions, particularly schizophrenia and bipolar disorder, due to their prevalence and significant financial impact in a public tertiary hospital setting. The high costs associated with treating schizophrenia and bipolar disorder underscore the need for ongoing research into more effective and affordable treatment options and for psychiatric disorders to be integrated more fully into the region’s growing evidence base on the economic burden of non-communicable disease.

## 1 Introduction

Non-communicable diseases (NCDs) are the leading cause of death worldwide, responsible for an estimated 41 million deaths annually and more than three-quarters globally, with over 75% of NCD deaths and 86% of premature NCD deaths (occurring between the ages of 30–70) recorded in low and middle-income countries (LMICs) (1). Age-specific NCD mortality in sub-Saharan Africa (SSA) already exceeds that of most other world regions, and NCDs are projected to overtake communicable, maternal, neonatal and nutritional diseases combined as the region’s leading cause of death by 2030, compounding health systems that remain oriented towards infectious disease control (2).

Psychiatric disorders occupy a distinctive position within this NCD burden. Although the World Health Organization (WHO) operational definition of the four major NCDs targeted for accelerated global action is limited to cardiovascular disease, cancer, diabetes and chronic respiratory disease (1) mental health is explicitly bound to the NCD agenda in global policy: Sustainable Development Goal (SDG) target 3.4 jointly commits to reducing premature NCD mortality by one third and to promoting mental health and wellbeing (3), and WHO situates its mental health programmes within a combined Non-communicable Diseases and Mental Health cluster. Clinically and economically, psychiatric disorders share the features that make the major NCDs costly to treat in resource-limited settings: they are chronic, require indefinite pharmacological management, demand regular clinical and laboratory monitoring, and expose patients and health systems to the risk of catastrophic or unaffordable expenditure (4, 5).

The prevalence of psychiatric conditions in adults varies globally, shaped by socioeconomic status, access to psychiatric health services, and cultural attitudes toward psychiatric wellbeing (6). In 2019, an estimated 970 million people globally (1 out of 8) were living with mental health challenges (7), and the inclusion of mental health among SDGs reflects growing recognition of its centrality to development (3, 6). A 2020 global study identified anxiety, depression, and bipolar disorder as the most prevalent psychiatric conditions, with schizophrenia the least prevalent (7).

In South Africa, the burden of psychiatric illness is substantial and rising; one in three South Africans is expected to experience a mental disorder in their lifetime, with a higher lifetime prevalence than in many other LMICs (8, 9). Substance use and addiction contribute substantially to the development of psychiatric conditions in South Africa (10), with chronic drug abuse is linked to serious psychiatric conditions, including generalised anxiety disorder, depression, bipolar disorder, and schizophrenia (10). Symptom severity varies among individuals (11), and many people who access psychiatric help can manage their symptoms and lead productive lives (12).

More than 75% of individuals in LMICs do not receive treatment for their psychiatric conditions despite the availability of effective, recognised treatments for psychiatric conditions (12), and scarce funding for mental health services remains a major barrier to adequate care in developing nations (7). The treatment gap is not confined to service availability; it is compounded by the financial cost of care to patients, families and health systems, a dimension shared with four major NCDs but comparatively under-quantified for mental disorders in SSA (13).

The consequences of psychiatric illness also extend beyond the patients and health system to the families who provide most day-to-day care. In Sub-Saharan Africa, where community-based mental services remain limited, family members commonly supervise treatment, accompany patients to clinics and meet care-related expenses, often at the expense of their own employment and income (13). A meta-analysis of 12 African studies estimated the pooled prevalence of caregiver burden at 61.7%, with higher burden among caregivers of people with schizophrenia (14), and a systematic review from Ethiopia found severe subjective burden among 63% of caregivers, with the heaviest burden among female caregivers, close relatives and those caring for people with schizophrenia-spectrum disorders or severe symptoms (15). This burden translates into poorer quality of life (QoL). In a study of 402 caregivers of psychiatric patients in two Ethiopian tertiary hospitals, 76.9% had poor overall QoL on the WHOQOL-BREF, with the social domain most severely affected (16), while caregivers in Eastern Ethiopia recorded their lowest score in the social domain, with caregiver depression, perceived stigma and perceived burden associated with worse QoL, and social support and resilience with better QoL (17). Similar patterns are evident in South Africa, where 44.7% of informal caregivers of people with severe mental disorders in rural Mpumalanga reported moderate to severe objective burden, particularly women, older caregivers and those living with the care recipient (18). Costs incurred by health systems, households and caregivers are therefore closely linked. When public services are inefficient or inaccessible, the burden of care, together with its financial and QoL consequences, shifts to families.

Psychiatric disorders contribute significantly to the global disease burden (6). The concept of burden of disease (BOD), also known as cost of illness (COI), encompasses various facets of disease impact on health outcomes at different levels-nationally, regionally, community, and individually (19, 20). Historically, COI studies categorised costs into direct, indirect, and intangible costs (21).

Beyond the immediate clinical burden, the cost of treating NCDs has emerged as a defining health systems challenge across LMICs, with direct implications for patients’ access to medicines. Cost of illness evidence indicates that annual per patient or per household costs of NCD care in LMICs are substantial and highly variable by condition: a systematic review of 41 quantitative cost studies estimated mean annual cost (in 2018 US dollars) of US$7,387 for chronic obstructive diseases, US$6,056 for cardiovascular disease, US$3,304 for cancer and US$1,017 for diabetes per patient or household (4). In another systematic review confined to Sub-Saharan Africa (SSA), it was similarly found that out-of-pocket (OOP) payments for NCD care frequently push households into catastrophic health expenditure (CHE), with reported prevalence for NCD care varying widely across the region and consistently higher among rural, poorer and uninsured households (5). Systematic reviews of the determinants of CHE in SSA corroborate this pattern, identifying chronic illness, rural residence, low socioeconomic status, older age and absence of health insurance as consistent risk factors for catastrophic OOP (22). While country-level studies from Ethiopia and Tanzania report that NCD multimorbidity and non-medical or opportunity costs (transport, lost income, caregiving time) substantially compound the direct medical cost burden of chronic disease care (23, 24).

A central driver of this financial burden is the persistent gap between the availability and affordability of essential medicines in the public and private sectors of LMICs. Using WHO/Health Action International survey methodology, a baseline assessment found that only 15.2% and 18.9% of the lowest-priced generic NCD medicines met WHO’s target of simultaneous availability and affordability in the public and private sectors of low-income countries, respectively, well short of WHO’s voluntary target of 80% availability of affordable essential NCD medicines by 2025 (25). Strikingly, a scoping review published in the same year that this WHO target expired found the same constraints essentially unchanged: availability of NCD medicines at the point of service delivery remained limited by facility type, supply chain inefficiencies, out-of-pocket payment requirements and weak procurement systems across LMICs (26), suggesting that nearly a decade of policy commitment to essential medicine affordability has outpaced actual implementation. These access barriers are not confined to the four major NCDs: the price of insulin, for example, has been reported to vary more than 30-fold between neighbouring African countries, reflecting weak price regulation and fragmented procurement rather than differences in clinical need (27).

South Africa illustrates both the scale of this challenge and possibilities for its mitigation. Systematic reviews of NCD intervention costs in the South African public sector show direct medicine and treatment costs for hypertension, type 2 diabetes and cardiovascular disease vary considerably depending on disease severity and complications, with a persistent shortage of public sector costing evidence relative to private sector data (28, 29). As South Africa moves towards National Health Insurance (NHI), robust, setting-specific costing evidence will be essential to inform the essential medicines list, provider reimbursement and resource allocation decisions that determine whether patients can access the medicine they need (10).

Despite the growing evidence base on the economic burden of the four major NCDs, mental disorders remain comparatively under-costed in the Sub-Saharan Africa costing studies, even though their aggregate economic burden is far from trivial. A recent macroeconomic modelling analysis projected that depression alone will impose a $375 billion economic burden on SSA between 2025 and 2050, equivalent to 0.493% of the region’s annual gross domestic product and around US$229 per capita, a lower absolute burden than high-income regions, but a comparable share of GDP, reflecting the region’s smaller economic base rather than smaller disease burden (30). This top-down, macroeconomic estimate stands in contrast to bottom-up, facility or system-level costing evidence available for South Africa specifically: narrative reviews of the South African mental health economics literature conclude that direct and indirect data for psychiatric, neurological and substance-abuse disorders remain sparse relative to their disease burden (9), and that where South African COI data for psychiatric disorders do exist they derive predominantly from national or health system-level expenditure (10) rather than the granular facility level, patient-costed studies more routinely available for hypertension, diabetes and cardiovascular diseases (28, 29). The two approaches are not directly comparable. One model aggregates productivity loss, the other itemises provider expenditure (31), but their divergence illustrates precisely the evidence gap this study addresses: a shortage of the kind of patient-level costing data for psychiatric disorders that is comparatively well established for the four major NCDs in South Africa.

In resource-limited settings, it is therefore essential for policymakers, health funders and hospital administrators responsible for budget allocation and decision-making (10, 11). This study aims to assess the direct healthcare costs of treating selected psychiatric conditions (bipolar disorder, schizophrenia, depression, and anxiety disorders) from the public sector provider’s perspective at a tertiary academic hospital in South Africa.

## 2 Methodology

### 2.1 Study site

The research was conducted at a public tertiary hospital situated in the north of Pretoria, South Africa, at the outpatient psychiatric clinic unit. The hospital is managed by the Department of Health and serves as a teaching hospital with specialist physicians and nurses. The hospital is funded by the government, which helps achieve the study’s objective of determining the cost of psychiatric treatment to assist care funders.

### 2.2 Study sample

The study population consisted of outpatient records for individuals aged 18 years and older who had been receiving treatment for selected psychiatric conditions, namely bipolar disorder, schizophrenia, depression, and anxiety disorders (*n* = 278), for a minimum duration of 12 months prior to data collection. We selected these conditions because they represented the predefined diagnostic groups among patients receiving long-term treatment at the study clinic and enabled diagnosis-specific treatment cost analysis. Diagnoses were based on those recorded in patients’ medical records by the treating clinicians and were not reassessed during the study. Recorded comorbidities, including hypertension, HIV and multimorbidity, were also extracted from the medical records. The analysis represents the treatment patterns recorded among eligible patients attending the selected tertiary hospitals’ outpatient psychiatric clinics. Prescribing within South African public-sector hospitals is guided by the applicable Adult Hospital Level Standard Treatment Guidelines and Essential Medicines List (STGs/EML) issued by the National Department of Health (32). The medicines actually prescribed and dispensed to patients were recorded; however, the study did not formally assess individual prescribers’ adherence to each STG/EML recommendation.

These patients were treated at the psychiatric clinic of an academic hospital in Pretoria during the period from February 2023 to February 2024. Stratified random sampling was utilised, in which the population was stratified into four subgroups according to the selected psychiatric condition. All patient files from the tertiary public hospital’s psychiatric clinic were requested from healthcare workers while they attended to the patient.

### 2.3 Study design

This retrospective descriptive, provider-perspective micro-costing study was based primarily on a review of patient medical records to estimate direct costs of the selected psychiatric conditions. The retrospective record review was supplemented by a prospective time-and-motion observation of the outpatient service. This prospective component was limited to observing and estimating the time healthcare workers and patients spent during outpatient psychiatric services. Participants were not followed prospectively as a cohort to assess clinical outcomes. We applied the average observed staff and service time for each condition when estimating administrative and labour costs.

The study used a bottom-up micro-costing approach to identify, quantify, and value the individual healthcare resources consumed in treating each patient (33, 34) All the costs were calculated to reflect the present-day cost of care in 2024.

### 2.4 Data collection and instrument

A three-month data collection cycle spanned a 12-month period from February 2023 to February 2024, while the extraction of patient-level data and the prospective time-and-motion observations were conducted between February and April 2024. To validate the accuracy of the data collection sheet, the researcher conducted a pilot study with five patients. To ensure that the data collection sheet was complete and appropriate to meet the study’s goals and objectives, and to answer the research question, the data collection sheet was then modified with contributions from the research supervisors. The data collection process involved recording demographic details (gender, age, race), diagnosis, and a list of medications prescribed for specific psychiatric conditions. This was done for all the patients who met the inclusion criteria.

Following this, the costs of each medication or supportive therapy were carefully documented. The direct cost of psychiatric treatment for each patient was calculated using micro-costing and bottom-up costing methods. The bottom-up approach provided an in-depth overview of activities and input usage derived from medical records or direct observation to estimate costs (26, 35).

The unit costs were obtained from relevant South African public-sector sources. Psychiatric and supportive medicine prices were obtained from the National Department of Health (NDoH) and the 2024 Master Health Product List (MHPL). Laboratory investigation costs were obtained from the National Health Laboratory Service 2023/2924 price list. Supportive therapy, such as Cognitive Behavioural Therapy (CBT) and counselling, was presented on separate days from medical consultations and medication collection; therefore, the time-in-motion method was not applied. These therapies were conducted either three or six times a year.

The costs for these services were as follows: sessions conducted by a psychiatrist (specialist medical provider) were charged at R319 per session, while sessions with a social worker or psychiatric nurse (allied supplementary health providers) were charged at R70 per session. These costs were sourced from the UPFS Gazette for Gauteng Province (2024) and were used to calculate the cost of supportive therapy per condition and per patient, as presented in Tables 1, 2, respectively. However, a time-in-motion method was employed to determine the time and cost involved in providing pharmacological treatment to outpatient psychiatric patients, with the average time applied retrospectively to all patients.

**Table 1:** Patients with antipsychotic injections in their prescriptions (*n* = 62).

Tasks	Average time taken with a health professional time (minutes and seconds converted to decimal number)	Average rate per health professional (*R*)	Average cost per patient (*R*)
Administration (preparation of file and checking of vitals by a nurse)	5 min 5 s (5.09)	2.44	12.42
Doctor consultation	15 min 7 s (15.11)	6.60	99.71
Nurse administering injection	7 min 6 s (7.10)	2.44	17.33
Pharmacist	8 min 6 s (8.10)	4.33	35.07
Total per patient per month	35 min, 24 s (8.10)	13.37	164.53
Total per patient per year			1,974.36

**Table 2:** Patients without injections in their prescriptions (*n* = 216).

Tasks	Average time taken with a health professional (minutes and seconds converted to decimal number)	Average rate per health professional (*R*)	Average cost per patient (*R*)
Administration (preparation of file and checking of vitals by a nurse)	5.09	2.44	12.42
Doctor consultation	15.11	6.60	99.71
Pharmacist	8.10	4.33	35.07
Total per patient per month	28.3 min	13.37	147.20
Total per patient per year			1,766.40

The costs of laboratory tests were calculated based on the tests each patient received, according to the National Health Laboratory Service (NHLS) list of laboratory tests and price reports for 2023/2024. Administrative costs, including healthcare worker salaries, were sourced from the Department of Health healthcare workers advertisements and the UPFS Gazette Annexure (2024).

The data collection instrument, adapted from a previously validated costing instrument (34), facilitated the systematic gathering of information, including demographics, psychiatric medication costs, laboratory tests, administrative costs, and supportive care medication costs. Demographic data, such as age, gender, ethnicity, treatment duration, diagnosis, and comorbidities, were obtained from patient medical records. Patient confidentiality was strictly maintained, and no personal identifying information, such as names or home addresses, was included on the data collection sheet.

### 2.5 Data entry and analysis

The data were captured using Microsoft Excel® spreadsheets, checked for accuracy, and cleaned before analysis. Data were analysed using the Statistical Package for the Social Sciences (SPSS) version 26, and all costs were expressed in Rands.

All data about demographics, medicine costs, administration costs, and supportive care costs were calculated and summarised as frequencies and means (averages). The costs of treating psychiatric conditions were calculated from the provider’s perspective and centred on the provider’s expenditures.

For recurring items, the quantities consumed were multiplied by the unit price to obtain the overall cost. For staff costs, an apportioning statistic based on the proportion of time devoted to treatment services related to both treatment arms was computed. The principal cost categories were medicines, laboratory and diagnostic investigations, staff and administrative services, consumables and supportive therapy.

The per-patient per-year (PPPY) costing model used an approach in which the total cost of treating a patient’s psychiatric condition over a year was calculated. This included all medical services, medications, and other relevant expenses. The costs associated with psychiatric treatment were calculated by multiplying each drug’s monthly cost by the 12-month review period.

Cost calculations were conducted using the following formulae:

*Total Medicine Cost*: Total Medicine Cost = Total Drug Cost × 12 (Monthly Visits to the Hospital for Collection)

*Total Administrative Cost*: Total Administrative Cost = Total Administration Cost per Visit × 12 (Monthly Visits).

*Total Treatment Cost per Month*: Total Treatment Cost per Month = Psychiatric Medication Cost + Administrative Cost + Supportive Psychotherapy Cost (e.g., Cognitive Behavioural Therapy)

*By multiplying the figures by 12, it was assumed that all patients adhered to therapy. This assumption simplified the calculations and made it easier to analyse the total costs according to the cost estimation assumptions studies (5).

### 2.6 Ethical considerations

Data collection commenced following approval from the university Research Ethics Committee, under clearance ID SMUREC/P/411/2023: PG, and authorisation from the hospital’s Chief Executive Officer. Since the researcher accessed patient files through healthcare personnel without direct interaction with patients, individual patient consent was not required. To protect the patient’s identity, each patient file was assigned a unique study number. To maintain confidentiality, only the study number was used as a reference in the database. The data collection forms did not include the patients’ identification numbers and names. All data extraction forms were coded, and no one else could access the patient information except the researcher. Any patient information was reported anonymously. Information gathered during and after the study will be kept in a locked cupboard in the supervisor’s office for 5 years and will then be destroyed by shredding. Similarly, information stored on computers is password-protected and is to be deleted 5 years after the study.

## 3 Results

Of the 278 patient files reviewed, 48.2% (*n* = 134) were male, and 51.8% (*n* = 144) were female, as demonstrated in Table 3. The majority of patients were Black, comprising 93.88% (*n* = 261) Age distribution showed that most patients were between 35 and 49 years old, accounting for 70.1% (*n* = 195) of the sample, with the peak age group identified as those aged 40–44 years, comprising 27.0% (*n* = 75). In terms of co-morbidity, the majority 72.3% (*n* = 201) of the patients had no co-morbid condition. A significant proportion of patients had comorbid conditions, with hypertension (6.5%) and HIV (9.4%) being the most common, particularly among those with schizophrenia. Multimorbidity, the presence of two or more chronic conditions in a single patient, was observed in 3.6% of the study population.

**Table 3:** Patient demographics (*n* = 278).

Demographics		Anxiety disorders *n* = 30	Bipolar disorder *n* = 81	Depression *n* = 61	Schizophrenia *n* = 106	Total *n* = 278
		*n* (%)	*n* (%)	*n* (%)	*n* (%)	*n* (%)
Gender	Male	6 (20.0)	29 (35,8)	7 (11.5)	92 (86.8)	134 (48.2)
	Female	24 (80.0)	52 (64.2)	54 (88.5)	14 (13.2)	144 (51.8)
Age group	18–24	1 (3.3)	1 (1.2)	3 (4.9)	2 (1.9)	7 (2.5)
	25–29	1 (3.3)	1 (1.2)	1 (1.6)	4 (3.8)	7 (2.5)
	30–34	2 (6.7)	16 (19.8)	4 (6.6)	10 (9.4)	32 (11.5)
	35–39	3 (10.0)	29 (24.7)	16 (26.2)	22 (20.8)	61 (21.9)
	40–44	11 (36.7)	19 (23.5)	12 (19.7)	33 (31.1)	75 (27.0)
	45–49	6 (20.0)	18 (22.2)	17 (27.9)	18 (17.0)	59 (21.2)
	50–54	4 (13.3)	5 (6.2)	5 (8.2)	10 (9.4)	24 (8.6)
	55–59	2 (6.7)	1 (1.2)	2 (3.3)	6 (5.7)	11 (4.0)
	>60	0 (0.0)	0 (0.0)	1 (1.6)	1 (0.9)	2 (0.7)
Co-morbidity	Hypertension	1 (3.3)	7 (8.6)	6 (9.8)	4 (3.8)	18 (6.5)
	Diabetes	0 (0.0)	12 (14.8)	2 (3.3)	0 (0.0)	14 (5.0)
	Arthritis	0 (0.0)	0 (0.0)	1 (1.6)	0 (0.0)	1 (0.4)
	Epilepsy	0 (0.0)	6 (7.4)	0 (0.0)	0 (0.0)	6 (2.2)
	Migraine	0 (0.0)	0 (0.0)	2 (3.3)	0 (0.0)	2 (0.7)
	Ulcers	0 (0.0)	0 (0.0)	0 (0.0)	0 (0.0)	0 (0.0)
	HIV	2 (6.7)	11 (13.6)	9 (14.8)	4 (3.8)	26 (9.4)
	Multimorbidity	0 (0.0)	4 (4.9)	4 (6.6)	2 (1.9)	10 (3.6)
	None	27 (90.0)	41 (50.6)	37 (60.7)	96 (90.6)	201 (72.3)
Race	Black	27 (90.0)	77 (95.1)	55 (90.2)	102 (96.2)	261 (93.9)
	White	0 (0.0)	0 (0.0)	4 (6.6)	2 (1.9)	6 (2.20)
	Colored	3 (10.0)	4 (4.9)	2 (3.3)	2 (1.9)	11 (4.0)

Table 4 shows that each month, A total of 675 packs of psychiatric medicines were dispensed, at an estimated cost of R25,868.50 per month and R310,422.00 per year. Antipsychotics were the most frequently dispensed class (52.15%) and the largest contributor to medicine expenditure (40.37%), followed by mood stabilizers/antiepileptics, which accounted for 17.63% of packs and 39.26% of costs. Together, these classes contributed 79.63% of total medicine expenditure. The estimated average medicine cost was R93.05 per patient per month and R1,116.63 per patient annually.

**Table 4:** Psychiatric conditions, pharmacological treatment, and their cost (*n* = 278).

Therapeutic class	Medicines included	Packs dispensed per month	Percentage of packs dispensed (%)	Monthly cost (*R*)	Annual cost (*R*)	Percentage of total medicine cost (%)
Antipsychotics	Amisulpride, clozapine, flupenthixol, olanzapine, quetiapine, risperidone and zuclopenthixol	352	52.15	10,441.93	125,303.16	40.37
Mood stabilisers/antiepileptics	Carbamazepine and sodium valproate	119	17.63	10,157.11	121,885.32	39.26
Antihistamine/sedative	Promethazine	76	11.26	3,404.04	40,848.48	13.16
Benzodiazepines/anxiolytics	Alprazolam, clonazepam and lorazepam	24	3.56	904.83	10,857.96	3.50
Antidepressants	Amitriptyline, citalopram and fluoxetine	101	14.96	785.06	9,420.72	3.03
Non-benzodiazepine hypnotic	Zolpidem	3	0.44	175.53	2,106.36	0.68
Total	All psychiatric medicines	675*	100.00	25,868.50	310,422.00	100.00
Average cost per patient (*n* = 278)	–	–	–	93.05	1,116.63	–

*Total number of items (pack size of 30) dispensed per month is 675.

Of the 278 patients who met the inclusion criteria, 110 were on supportive medications (Table 5). Only three supportive medications were noted in patient files: Orphenadrine 50 mg for 52 patients (notably 31 schizophrenia and 21 bipolar patients), Folic Acid 5 mg for 48 bipolar patients (particularly females on sodium valproate), and Propranolol 10 mg for 10 anxiety patients. Orphenadrine contributed the highest cost at R25,958.4 annually, followed by Folic Acid (R2,937.600).

**Table 5:** Cost of supportive medications (including medications for treating side effects; *n* = 110).

Supportive care medications	Number of patients	Cost per item (rands)	Total amount per month (rands)	Total amount per year (rands)
Orphenadrine 50 mg	52	41.6	2,163.2	25,958.40
Folic acid 5 mg	48	5.1	244.80	2,937.60
Propranolol 10 mg	10	5.43	54.30	651.60
Total for both items			2,462.30	29,547.00

Table 6 shows the costs associated with laboratory, diagnostic and radiological tests for psychiatric patients (*n* = 278), most of which were conducted twice a year. Blood was drawn for all tests, charged at R49 per patient per blood drawing procedure. This procedure was performed twice yearly, as all laboratory tests occurred biannually. CMPU, electrolytes and full blood count (FBC) tests contributed the most to the total expenditure, as they were performed on all patients twice a year. The liver function test (LFT) was the most expensive, at R426 per test, but was conducted in a few patients (only 21 out of 278 patients). Diagnostic and radiological tests and positron emission tomography (PET) scans were the most costly items overall (R14,190 and R10,215 per patient per test, respectively), though these tests were required less frequently.

**Table 6:** Cost of laboratory tests for all patients (*n* = 278).

Laboratory test	Cost per test in rands®	Number of times the test was conducted per year	Anxiety disorders (*n*)	Bipolar disorder (*n*)	Depression (*n*)	Schizophrenia (*n*)	Total patients	Total cost in rands per year (*R*)	Average cost in rands per patient per year (*R*)
TCA level	113.01	2	0	2	2	0	4	904.08	226.02
VPA level	113.01	2	0	54	0	22	76	17,177.52	226.02
BDZ level	113.01	2	2	4	12	3	21	4,746.42	226.02
Carbamazepine level	113.01	2	0	4	0	0	4	904.08	226.02
Clozapine level	113.01	2	0	0	0	3	3	678.06	226.02
FBC tests	89.55	2	30	81	61	106	278	49,789.80	179.10
LFT tests	426	2	2	8	6	5	21	17,892.00	852.00
CMPU tests	91.05	2	30	81	61	106	278	50,623.80	182.10
Other electrolytes and creatinine	105.15	2	30	81	61	106	278	58,463.40	210.30
PET-scan	14,190	1	1	1	0	0	2	28,380.00	14,190.00
MRI scan	10,215	1	0	1	0	1	2	20,430.00	10,215.00
CT-scan	4,496	1	0	0	1	0	1	4,496.00	4,496.00
ECG	351	1	0	1	2	0	3	1,053.00	351.00

Mean = 1,993.36; SD = 9,223.372; *p*-value < 0.001.

BDZ, Benzodiazepines; CMPU, Creatinine, Magnesium, Phosphate, and Urea; CT, Computed Tomography; ECG, Electrocardiogram; FBC, Full Blood Count; LFT, Liver Function Test; MRI, Magnetic Resonance Imaging; PET, Positron Emission Tomography; TCA, Tricyclic Antidepressants; VPA, Valproic Acid.

Table 7 presents the cost of consumables used per patient visit for 62 patients (consisting of bipolar disorder and schizophrenia patients) receiving monthly intramuscular injections of either Zuclopenthixol or Flupenthixol. Consumable use was consistent across all 62 patients regardless of condition. Gloves were the cost driver among the consumables, costing R12.24 per patient annually, and alcohol swabs cost the least, at R1.20 per patient per year.

**Table 7:** Cost of consumables (*n* = 62).

Consumables	Number of items per patient per visit/during the study period	Cost per item (rands)	Total Cost of items used for all patients per month (rands)	Total Cost of items used for all patients per year (rands)	Average cost per patient per year (rands)
Gloves non-sterile, single-use	1	1.02	63.24	758.88	12.24
22G x 50”mm needle	1	0.45	27.90	334.80	5.40
5 mL syringe	1	0.62	38.44	461.28	7.44
Alcohol swab	1	0.1	6.20	74.40	1.20
Total for all items			135.78	1,629.36	26.28

Sixty-two patients had injections included in their prescriptions, while 216 patients did not. The labour cost per patient per year for those who received injections was R1,974.36 (Table 1), compared with R1,766.40 for those who did not (Table 2).

The total annual cost for the 278 patients was R1,672,998.84. Schizophrenia had the highest mean annual cost per patient at R7,248.25, followed by bipolar disorder (R5,762.10), anxiety disorders (R4,853.14) and depression (R4,792.79). Compared with depression, mean costs were R2,455.46 higher for schizophrenia, R969.31 higher for bipolar disorder and R60.35 higher for anxiety disorders. The main cost drivers were laboratory, diagnostic and radiological testing for anxiety disorders (41.4%) and bipolar disorder (34.8%), labour and administrative services for depression (36.9%), and supportive care therapies for schizophrenia (36.7%; Table 8).

**Table 8:** Comparison of average total cost per year by diagnosis.

Diagnosis	Cost component	Annual cost (*R*)	Average cost per patient (*R*)	Contribution (%)	Interpretation
Anxiety disorders (*n* = 30)	Psychiatric medications	12,412.80	413.76	8.5	
	Supportive medications	998.40	33.28	0.7	
	Laboratory, diagnostic and radiological tests	60,291.04	2,009.70	41.4	Largest cost driver
	Consumables	0.00	0.00	0.0	No recorded cost
	Labour/administrative service time	52,992.00	1,766.40	36.4	
	Supportive care therapies	18,900.00	630.00	13.0	
	Total	145,594.24	4,853.14	100.0	
Bipolar disorder (*n* = 81)	Psychiatric medications	101,746.08	1,256.12	21.8	
	Supportive medications	6,757.20	83.42	1.4	
	Laboratory, diagnostic and radiological tests	162,478.78	2,005.91	34.8	Largest cost driver
	Consumables	183.96	2.27	0.0	
	Labour/administrative service time	144,534.12	1,784.37	31.0	
	Supportive care therapies	51,030.00	630.00	10.9	
	Total	466,730.14	5,762.10	100.0	
Depression (*n* = 61)	Psychiatric medications	39,003.36	639.40	13.3	
	Supportive medications	5,990.40	98.20	2.0	
	Laboratory, diagnostic and radiological tests	101,185.78	1,658.78	34.6	
	Consumables	0.00	0.00	0.0	No recorded cost
	Labour/administrative service time	107,750.40	1,766.40	36.9	Largest cost driver
	Supportive care therapies	38,430.00	630.00	13.1	
	Total	292,359.94	4,792.79	100.0	
Schizophrenia (*n* = 106)	Psychiatric medications	93,294.36	880.14	12.1	
	Supportive medications	15,150.00	142.92	2.0	
	Laboratory, diagnostic and radiological tests	177,832.56	1,677.67	23.1	
	Consumables	1,445.40	13.64	0.2	
	Labour/administrative service time	198,676.20	1,874.30	25.9	
	Supportive care therapies	281,916.00	2,659.58	36.7	Largest cost driver
	Total	768,314.52	7,248.25	100.0	

## 4 Discussion

This study quantified the direct healthcare costs associated with anxiety disorders, bipolar disorder, depression and schizophrenia among 278 patients. The total annual cost was R1,672,998.84, with schizophrenia having the highest mean annual cost per patient (R7,248.25), followed by bipolar disorder (R5,762.10), anxiety disorders (R4,853.14) and depression (R4,792.79). Antipsychotics and mood stabilisers/antiepileptics together accounted for 79.63% of psychiatric medicine expenditure. However, the principal cost drivers extended beyond medicines: laboratory, diagnostic and radiological tests predominated for anxiety and bipolar disorders; labour and administrative services for depression; and supportive care therapies for schizophrenia. These findings demonstrate that, as with the four major NCDs, the cost of managing psychiatric conditions is shaped less by any single input than by the specific combination of monitoring medication and supportive services each diagnosis requires (4, 28).

In the study, schizophrenia was the major cost driver among psychiatric conditions, accounting for 38.1% of the total sample and incurring the highest total treatment costs. The high costs of schizophrenia outpatient management are consistent with the chronic, relapsing course of the disorder, which requires long-term pharmacological management, frequent laboratory monitoring, and intensive supportive care (36). Similarly, the high cost of managing bipolar disorder (the second highest cost driver) was attributable to the need for mood stabilisation and regular laboratory testing, consistent with findings from South Africa and Indonesia (10, 11). Injectable therapies resulted in higher resource demands because nurses had to administer the injections following a doctor’s consultation. The findings align with a study from the United States, which similarly reported higher short-term costs for injectable treatments, while noting potential long-term cost savings from improved adherence and reduced hospitalisations (28, 37).

Laboratory, radiological and diagnostic tests emerged as the largest cost drivers for several diagnoses, although the seemingly higher average cost of these tests among anxiety disorder patients was heavily influenced by a single outlier, a patient who underwent a PET scan, which likely skewed this subgroup’s results. While judicious use of laboratory, radiological and diagnostic tests can be invaluable for diagnosis, monitoring medication side effects, and guiding management decisions, inappropriate utilisation can inflate costs, lead to misinterpretations of symptoms, and expose patients to unnecessary procedures (38).

CMPU and Full Blood Count (FBC) tests were nonetheless essential for monitoring treatment safety and efficacy. CMPU tests assessed vital minerals to help prevent medication related side effects, while FBC tests are central to monitoring the metabolic and cardiovascular risk associated with atypical antipsychotics (39, 40). Monitoring Valproate levels (mood stabiliser) in bipolar disorder patients accounted for substantial costs, reflecting standard practice around maintaining therapeutic drug levels to prevent relapse and adverse events. Advanced diagnostic imaging (PET and MRI) incurred the highest per-test costs, while such modalities improve diagnostic accuracy; their expense warrants judicious use in resource-constrained public healthcare systems. This tension mirrors debates in the broader NCD literature about balancing diagnostic thoroughness against affordability (28, 29).

Set against the wider cost-of-illness literature on NCDs in LMCs, the medicine costs observed in this cohort are strikingly modest. The average psychiatric medicine cost per patient in this study was approximately R1,116.63 per year, roughly equivalent to US$60–65 at 2024 exchange rates, and is far below the mean costs reported in a systematic review of 41 LMIC cost of illness studies, which found per patient or per household NCD costs ranging from US$1,017 for diabetes to US$7,387 for chronic obstructive pulmonary diseases (4). It is likewise substantially lower than the medicine costs reported for the same psychiatric diagnoses in Indonesia (11), and the hypertension and type 2 diabetes drug costs reported in South Africa’s systematic review, which ranged from US$2 to US$85 per person per month for hypertension and US$ 57 to US$ 630 per person per year for type 2 diabetes (28). This discrepancy most plausibly reflects the public-sector subsidised nature of the setting studied here, consistent with an earlier national survey showing that South Africa’s public mental health system relies on a restricted, centrally procured essential medicines list rather than originator brand pricing (10). It also illustrates a broader point emerging from NCD medicines access literature where essential medicines are reliably available through the public sector and procured close to WHO’s affordability benchmarks, the direct cost of pharmacological treatment can remain low even for conditions such as schizophrenia and bipolar disorder that require multiple, sometimes co-prescribed, long-term medications (25, 26).

This comparison must nonetheless be read with caution. Because the present costing was conducted entirely from the public-sector provider perspective, it excludes the out-of-pocket, non-medical, and opportunity costs (transport, lost income, caregiving time) that dominate the NCD cost results from other SSA settings and that push households into catastrophic health expenditure (5, 22–24). Patients who face medicines stockouts, referral to private pharmacies, or travel to specialised centres for supportive therapy as required for schizophrenia management in this cohort may incur substantial costs not captured here. The true household-level cost of psychiatric care in South Africa’s public sector is therefore likely to be considerably higher than the provider-perspective estimates reported in this study, and future costing studies should incorporate the patient and household perspective to establish whether the same catastrophic expenditure risk documented for hypertension, diabetes and cardiovascular diseases elsewhere in SSA also applies to psychiatric conditions.

Comparison with psychiatric cost-of-illness from other settings helps place these findings in context. At an approximate 2024 exchange rate of 18.3 per US dollar, the mean annual provider cost per patient in this study was about US$395 for schizophrenia, US$315 for bipolar disorder and US$260–265 for depression and anxiety disorders. In a Nigerian teaching hospital, the direct cost of outpatient schizophrenia care was US$349,59 per patient per year, of which medicines accounted for 51.2% and hospitalisation for 21.0%, with a further US$468.89 in indirect costs, mainly lost productivity and transport (41). A second Nigerian study found that 96% of psychiatric outpatients financed their treatment themselves or through their families, and that 12% had already incurred catastrophic health expenditure (42). In Brazil’s public health system, the mean annual cost of treating schizophrenia with atypical antipsychotics was US$1.811.92 per patient, with atypical antipsychotics alone accounting for almost 80% of costs (43). Globally, a systematic review of 143 cost-of-illness studies reported a median societal cost for schizophrenia of US$13,256 (purchasing power parity) per patient per year, the highest of the mental disorders with adequate data, with almost half of total costs attributable to indirect costs. Notably, only three of the included studies were from Africa (44). Differences in costing perspective, currency year, cost components and health system organisation limit direct comparison. Nevertheless, the ranking observed in the present study, with schizophrenia the most costly condition, is consistent with this international evidence (44), while the composition of costs differs markedly between settings: medicines dominate where atypical or originator products are widely used (43), and hospitalisation and patient-borne costs dominate where care is paid (41, 42).

Within South Africa’s public sector, where generic psychotropic medicines are centrally procured, the main outpatient cost drivers are instead monitoring, staff time and supportive care. The mean annual cost of care in this study was approximately R6,018 per patient (R1,672998.84 across 278 patients). Of this, psychotropic medicines accounted for R1,116.63 (about 19%), whereas labour costs alone amounted to R1,766.40-R1,974.36 per patient per year. Staff time was therefore a larger recurring expense than medicines, reflecting the monthly clinic required for medicine collection and, for the 62 patients on depot antipsychotics, the additional nursing time needed for injection administration. This has practical significance in a health system facing a severe shortage of mental health professionals, with only 0.31 psychiatrists per 100,000 uninsured population (10). Measures that reduce the number of facility visits for clinically stable patients without compromising monitoring may therefore yield greater efficiency gains than further reductions in medicines prices,

Supportive care, including CBT and counselling, played a crucial role in psychiatric management in this study, aligning with findings from a Swedish study on the effectiveness of CBT in routine psychiatric care (45). Costs varied significantly by provider type; specialist psychiatrists, primarily involved in schizophrenia management, had a higher consultation cost (R319) than therapists, social workers, and psychiatric nurses, classified as allied supplementary health treatment at a lower cost of R70 per session. This cost disparity has important implications for resource allocation, as directing lower acuity supportive care towards lower-cost providers could improve cost efficiency without compromising access to essential therapies, provided the availability of qualified allied health professionals is maintained (10).

Consumables contributed minimally to total costs in our study, consistent with a similar study by Jacobs and Coetzee (49), which also demonstrated that consumable item costs in psychiatric care are negligible compared to those in other medical specialities. While consumables contributed minimally to overall costs, supportive medications such as Propanolol, Orphenadrine, and Folic Acid also played a limited but important role in patient management in this current study.

The study provides insights into the significant burden of mental health conditions in South Africa, where psychiatric disorders rank as the third-highest disease burden after HIV/AIDS and other infectious diseases (10). This position within South Africa’s overall disease burden is consistent with global modelling suggesting that, while SSA’s absolute economic burden from depression is lower than in high-income regions, it represents a comparable share of GDP (approximately 0.49%) and a substantial per capita cost relative to the region’s income levels (30). Because psychiatric illness, particularly schizophrenia and bipolar disorder, frequently requires long-term family and community support, the economic burden documented here for the health system is likely accompanied by further, largely uncosted burden on family caregivers, consistent with systematic review evidence from SSA (13).

The provider costs reported here present only part of the wider economic and social costs of psychiatric illness. Globally, almost half of the total cost of mental disorders arises from lost productivity and other indirect costs rather than from health care costs (45), and 70.1% of patients in this study were aged 35–49 years, a period of peak economic and family responsibility. In the African context, much of the remaining burden is absorbed by family caregivers, whose own quality of life is substantially compromised. Severe caregiver burden affects around six in ten caregivers of people with mental illness in Africa and in Ethiopia (14, 15); poor overall QoL was reported by 76.9% of caregivers of psychiatric patients in an Ethiopian tertiary hospital (16); and caregiver burden, depression and perceived stigma have been associated with lower QoL across all domains (17). In South Africa, women, older caregivers and those living with the care recipient carry the heaviest objective burden (18). Because schizophrenia, the most costly condition to the provider in this study, is also consistently associated with the greatest caregiver burden and poorer caregiver QoL (14, 15, 17), provider-perspective estimates are likely to understate the full societal cost of the conditions studied. From a public health and economic perspective, however, the case for investment is favourable. A global return-on-investment analysis estimated that every US$ invested in scaling up treatment for depression and anxiety yields a return of US$2.0–3.0 in economic productivity alone, rising to US$3.3–5.7 when the value of health gains is included (46).

The gender distribution among the participants was nearly equal; however, a notable variation emerged when analysing gender distribution across specific psychiatric conditions. Depression was significantly more prevalent among females (88.5%) than males (11.5%), and anxiety disorders were similarly skewed (80% females, 20% males). Schizophrenia, in contrast, showed a predominance among males (86.8% versus 13.2% females). These findings align with research done in the United States of America in 2015, showing that women experience significantly higher rates of depression and anxiety in adulthood, while. Men are more likely to exhibit substance use disorders and antisocial behaviours (47). The highest prevalence of mental health conditions was observed among individuals aged 35–49 years, particularly in the 40–44 age group, highlighting the impact of these disorders on individuals in their most economically productive years. Although comorbid conditions were found in a small percentage, it still underscores the necessity for integrated care models that address both psychiatric and physical health challenges, given the known interplay between mental and physical health (47).

Understanding these key cost drivers is essential for informing pharmacoeconomic evaluations and resource allocation decisions, contributing to equitable and effective mental healthcare provision as South Africa progresses towards implementing NHI.

### 4.1 Implications for practice and policy

Five implications follow from the findings of our study, with relevance both to South Africa and to other low-income and middle-income settings. First, because psychiatric medication costs in this cohort were low only because essential psychotropic medicines were consistently available through public sector procurement, safeguarding the supply chain, rather than assuming it will persist by default, should be an explicit NHI and provincial health priority; the wider NCD literature shows how quickly low-cost, stable access can be undermined by stockouts, weak procurement and price fragmentation (25–27). Second, as with the four major NCDs, psychiatric medicines should be protected on the essential medicines list under NHI benefit design on the same basis as antihypertensives and antidiabetic medicines, rather than being treated as a lower priority; the comparable per-capita economic burden of depression alone in this region suggests the case for doing so is at least as strong (30).

Third, because laboratory, diagnostic and administrative/labour costs, not medicines, were the dominant cost-drivers for three of the four conditions studied, NHI benefit-package costing for psychiatric care should explicitly budget for routine monitoring (FBC, CMPU, anticonvulsant levels) and staff time, rather than assuming that a low medicine cost implies a low overall cost of care. Fourth, the cost differential between psychiatric-led and allied provider supportive therapy sessions observed here supports shifting routine counselling and CBT to trained social workers and psychiatric nurses where clinically appropriate, freeing psychiatric capacity for the more complex, medication-intensive management that schizophrenia and bipolar disorder require (10). This is provided that this is accompanied by investment in the supervision and training needed to maintain quality care (45).

Fifth, the findings point to several cost-containment and efficiency measures relevant to South Africa and other LMICs with comparable public-sector psychiatric services. (i) Rationalising investigations: routine monitoring should follow drug-specific, evidence-based protocols (for example, valproate levels and metabolic monitoring where indicated), while high-cost imaging such as PET and MRI should be reserved for clearly defined clinical indications through referral; (ii) Reducing visit-related staff costs: clinically stable patients could be given longer refill intervals and collect medicines through decentralised community-based dispensing, such as South Africa’s Central Chronic Medicines Dispensing and Distribution (CCMDD) programme, freeing clinic time for patients with greater needs; (iii) Task sharing and integration into primary care: evidence from the region shows that trained lay health workers can deliver effective psychological treatment for common mental disorders in primary care, as demonstrated by the Friendship Bench trial in Zimbabwe (48), supporting the management of stable patients with depression and anxiety outside tertiary institutions; (iv) Protecting continuity of care: because inpatient care accounts for most public mental health spending in South Africa (10) Adherence support and uninterrupted outpatient follow-up are likely to be among the most efficient uses of limited resources; and (v) Supporting families? Because social support and lower stigma are associated with lower caregiver burden and better caregiver QoL (15, 17)), caregiver psychoeducation and support could be offered alongside routine clinic visits at modest cost, recognising that sustaining family caregivers also helps sustain treatment.

### 4.2 Strengths and limitations

This study’s principal strength lies in its granular, facility-level micro-costing approach, which, unlike other NCD cost analyses that rely on national aggregate expenditure data (10, 28), itemises costs by treatment component and diagnosis, offering the kind of patient-level costing evidence identified as scarce for both psychiatric and non-communicable disease research in this setting (29).

This study has several limitations. Firstly, it focuses solely on direct medication costs for outpatient management and thus cannot be generalised, as these patients are often readmitted to hospital. The costs presented are averages and may not reflect individual variations in individual patient responses to medications, which can affect treatment choices and duration. Including only patients on treatment for at least a year excludes individuals in the early stages of treatment, who may incur higher initial costs due to diagnostic tests, medication titration, and frequent follow-up visits, potentially leading to an underestimation of the overall cost of care for newly diagnosed patients. Additionally, the research is based on a single public tertiary hospital in South Africa, which limits the generalisability of the findings to other settings or regions with different prescribing practices and resource availability.

Finally, because the analysis was restricted to the provider’s perspective, it does not capture the out-of-pocket, non-medical, or indirect costs shown in other studies to be a major, sometimes dominant component of the household burden of NCD care (5, 9, 23). Future costing studies of psychiatric care in this setting should incorporate the patient and household perspective to provide a fuller picture of the affordability of psychiatric care under NHI, comparable to household-level evidence already available for several major NCDs in SSA. Such studies also measure caregiver time and quality of life, given the substantial caregiver burden documented in the region (14–18).

## 5 Conclusion

This study provides a facility-level estimate of the direct healthcare costs associated with managing four psychiatric conditions in a South African public tertiary hospital. Mean annual costs varied by diagnosis, with schizophrenia having the highest cost per patient, driven largely by supportive care therapies, while diagnostic testing and labour-related services were important cost components across the other conditions. Medicines costs in the public sector setting were markedly lower than those reported for other major non-communicable diseases and for psychiatric conditions elsewhere in the region, underscoring both the value of a well-functioning essential medicines supply chain and the need for costing studies that also capture the out-of-pocket and indirect costs known to drive catastrophic expenditure among patients with chronic conditions across sub-Saharan Africa. As South Africa advances towards NHI, these findings may help inform budgeting, resource planning, and essential medicines list decisions for psychiatric services, and highlight psychiatric disorders as a category of non-communicable diseases requiring the same sustained costing attention already directed at cardiovascular diseases, diabetes, and cancer.

## Data Availability

The raw data supporting the conclusions of this article will be made available by the authors, without undue reservation.
